# Antamanide, a Derivative of *Amanita phalloides*, Is a Novel Inhibitor of the Mitochondrial Permeability Transition Pore

**DOI:** 10.1371/journal.pone.0016280

**Published:** 2011-01-28

**Authors:** Luca Azzolin, Nicola Antolini, Andrea Calderan, Paolo Ruzza, Marco Sciacovelli, Oriano Marin, Stefano Mammi, Paolo Bernardi, Andrea Rasola

**Affiliations:** 1 Department of Biomedical Sciences University of Padova and CNR Institute of Neuroscience, Padova, Italy; 2 Department of Chemical Sciences, University of Padova and CNR Institute of Biomolecular Chemistry, Padova, Italy; 3 Department of Biological Chemistry, University of Padova, Padova, Italy; 4 Venetian Institute of Molecular Medicine, University of Padova, Padova, Italy; Instituto de Química - Universidade de São Paulo, Brazil

## Abstract

Antamanide is a cyclic decapeptide derived from the fungus *Amanita phalloides*. Here we show that antamanide inhibits the mitochondrial permeability transition pore, a central effector of cell death induction, by targeting the pore regulator cyclophilin D. Indeed, (i) permeability transition pore inhibition by antamanide is not additive with the cyclophilin D-binding drug cyclosporin A, (ii) the inhibitory action of antamanide on the pore requires phosphate, as previously shown for cyclosporin A; (iii) antamanide is ineffective in mitochondria or cells derived from cyclophilin D null animals, and (iv) abolishes CyP-D peptidyl-prolyl *cis-trans* isomerase activity. Permeability transition pore inhibition by antamanide needs two critical residues in the peptide ring, Phe6 and Phe9, and is additive with ubiquinone 0, which acts on the pore in a cyclophilin D-independent fashion. Antamanide also abrogates mitochondrial depolarization and the ensuing cell death caused by two well-characterized pore inducers, clotrimazole and a hexokinase II N-terminal peptide. Our findings have implications for the comprehension of cyclophilin D activity on the permeability transition pore and for the development of novel pore-targeting drugs exploitable as cell death inhibitors.

## Introduction

Antamanide (AA) is a monocyclic, homodetic decapeptide isolated from the poisonous mushroom *Amanita phalloides* [1]. AA has been extensively studied for its strong antidote activity against phallotoxins and amatoxins, which are extremely toxic peptides isolated from the same fungus [2]. It was also reported that AA inhibits tumor cell growth *in vitro* [3], displays an antitumor action in an animal model [4], and attenuates IL-2-induced multisystem organ edema [5]. Little is known about the biochemical mechanisms underlying these diverse bioactivities. To explain its antitoxic activity, it was proposed that AA competitively antagonizes a hepatocyte membrane transporter for the phallotoxin phalloidin and for the amatoxin alpha-amanitin [6], [7]. This transporter was later identified as a member of the organic anion-transporting polypeptide family [8], [9]. Notably, cell uptake of phalloidin was also inhibited by the immunosuppressive drugs rapamycin, FK506 or cyclosporin A (CsA) [8], and AA itself acts as an immunosuppressant [10], [11]. These observations strongly suggest that AA could interact with the immunophilins FK506BP or cyclophilin (CyP) A, which are the protein targets of rapamycin/FK506 and CsA, respectively [12], [13].

CyP-A is a component of the CyP protein family, whose members display peptidyl-prolyl *cis-trans* isomerase activity [14] and are characterized by a high degree of sequence conservation and by a differential subcellular distribution [15]. We therefore reasoned that if the AA target was the cytosolic CyP-A, the drug could also act on other members of this protein family. Indeed, such a pleiotropic effect is well-characterized for CsA, as CsA also targets the mitochondria-restricted CyP-D [16]–[18]. CyP-D displays an important role in the cell response to a variety of noxious stimuli, as it modulates a channel located in the inner mitochondrial membrane, the permeability transition pore (PTP) [19], [20], whose prolonged opening irreversibly commits cells to death [21]. PTP dysregulation is emerging as a common feature in a variety of pathologies endowed with either an excess of cell death, such as neurodegenerative disease or muscular dystrophies, or with an aberrant hyperactivation of survival pathways, as in cancer [21], [22]. CsA inhibits PTP opening through binding to CyP-D [21]. Therefore, it constitutes an interesting molecule for the treatment of degenerative diseases [23], [24]. Nonetheless, due to its immunosuppressant activity, to its side effects [25] and to its inability to pass the blood-brain barrier [24], CsA analogues with a higher selectivity for CyP-D are under intense scrutiny [23], [26]–[29].

Here we demonstrate that, similar to CsA, AA targets CyP-D leading to PTP inhibition and to cell protection from insults that cause pore opening. AA could be exploited as a lead compound for a new class of PTP-inhibiting drugs.

## Results

### AA inhibits the PTP in isolated mitochondria

**Figure 1:**
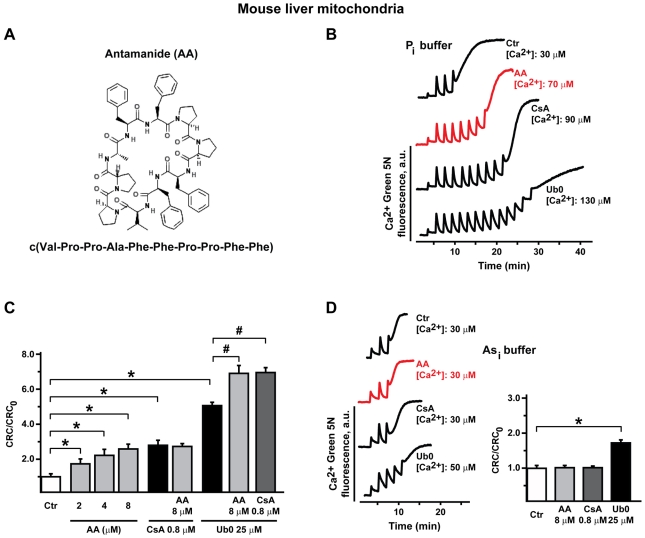
Effect of AA on PTP opening in isolated mouse liver mitochondria. A, chemical structure of AA. B, D, Ca^2+^ retention capacity (CRC) either in phosphate (P_i_) buffer (B) or in arsenate (As_i_) buffer (D). Calcium Green-5N fluorescence is reported as arbitrary units on the *y* axis. As the probe does not permeate mitochondria, Ca^2+^ uptake into the organelles is displayed as a rapid decrease of the fluorescence spike after administration of every Ca^2+^ pulse (10 µM each). AA (red trace, 8 µM) or CsA (0.8 µM) act as pore inhibitors only in P_i_ buffer (B), as they increase the threshold Ca^2+^ concentration required to trigger the permeability transition, *i.e.* the number of spikes before a sudden and marked fluorescence increase occurs. Ub0 (25 µM) inhibits the pore also in As_i_ buffer, albeit to a lesser extent. C, inset of D, quantification of the effect of PTP inhibitors is displayed as the ratio between the CRC detected in the presence (CRC) and absence (CRC_0_) of the compound. Results are mean±SD of at least 4 experiments. In C and D, we analyzed whether each pharmacological treatment increased mitochondrial Ca^2+^ uptake when compared to control conditions (Ca^2+^ uptake in the absence of the drug), and found a significant difference (Student's *t* test analysis; *: p<0.01) between the CRC of mitochondria treated with either AA (at various concentrations), or CsA, or Ub0 and the CRC of untreated mitochondria, indicating that each of these treatments inhibits the PTP. In C, significant differences were also observed between the CRC of mitochondria treated with either Ub0/AA or Ub0/CsA and with Ub0 by itself (Student's *t* test analysis; #: p<0.01), indicating that the inhibitory effect of both AA and CsA on the PTP is additive with that of Ub0.

**Figure 2:**
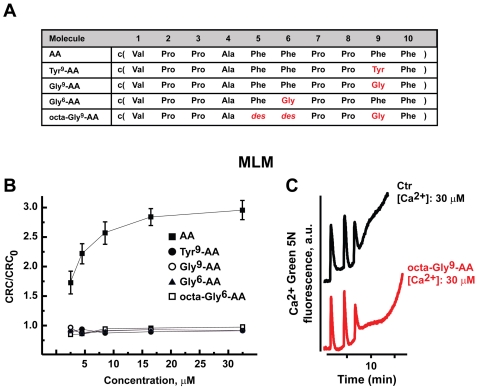
Phe residues in position 6 and 9 of AA are required for PTP inhibition. A, amino acid sequence of AA derivatives tested on the PTP. Amino acid changes are outlined in red. B, Ratio between the CRC detected in the presence (CRC) and absence (CRC_0_) of increasing concentrations of AA and derivatives. The CRC/CRC_0_ ratio is calculated as in Figure 1. Results are mean±SD of at least 4 experiments. C, Representative experiment showing the lack of effect on Ca^2+^ retention capacity (CRC) of the octa-Gly^9^ AA derivative. Traces are reported as in Fig. 1B.

AA is the cyclodecapeptide c(Val-Pro-Pro-Ala-Phe-Phe-Pro-Pro-Phe-Phe) (Figure 1A). To evaluate its effect on the PTP, we performed Ca^2+^ retention capacity (CRC) assays on isolated mouse liver mitochondria (MLM). Notably, when mitochondria were incubated in a phosphate-containing medium, AA inhibited pore opening, similar to the PTP inhibitors CsA or Ubiquinone 0 (Ub0; Figure 1B,C). PTP inhibition by AA was not additive with that of CsA, whose molecular target is CyP-D, while AA did increase inhibition by Ub0, which is independent of CyP-D (Figure 1C). We had shown that the effect of CsA, but not of Ub0, is abolished by substituting phosphate with arsenate [30]. Likewise, AA inhibition of the PTP was abrogated in the presence of arsenate (Figure 1D). To dissect AA potency as a PTP inhibitor and the residues involved in its activity, we performed a concentration-response CRC experiment on MLM treated with AA or with a panel of derivatives (Figure 2A). We found that the effect of AA reached a plateau at a concentration of about 20 µM, and that changing amino acids in position 6 or 9 completely abolished pore inhibition (Figure 2B,C).

### CyP-D is the molecular target of AA for PTP regulation

**Figure 3:**
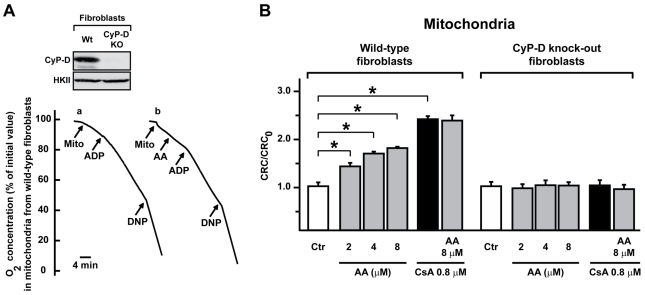
Effect of AA on respiration and PTP opening in mitochondria of wild-type or CyP-D knock-out mouse fibroblasts. A, upper part: Western blot analysis assessing CyP-D expression in fibroblasts obtained from either wild-type or CyP-D knock-out mice; HKII was utilized to verify protein load; lower part: representative experiment showing oxygen consumption as assessed by polarography (see Methods) in mitochondria isolated from wild-type fibroblasts in the absence (a) or presence (b) of 8 µM AA. Experiments were started by adding 0.6 mg of mitochondria (Mito), followed by 100 µM ADP and 50 µM 2,4-dinitrophenol (DNP) (arrows). Each experiment was repeated at least three times. Oxygen consumption is reported as percentage of initial value on the *y* axis. B, bar graphs report the ratio between the CRC detected in the presence (CRC) and absence (CRC_0_) of increasing concentrations of AA, in mitochondria from either wild-type fibroblasts (left) or CyP-D knock-out fibroblasts (right). PTP inhibition by AA and CsA is not additive. Results are mean±SD of at least 4 experiments. We analyzed whether each pharmacological treatment increased mitochondrial Ca^2+^ uptake when compared to control conditions (Ca^2+^ uptake in the absence of the drug), and found a significant difference (Student's *t* test analysis; *: p<0.01) between the CRC of mitochondria treated with either AA (at various concentrations), or CsA, and the CRC of untreated mitochondria, indicating that each of these treatments inhibits the PTP.

**Figure 4:**
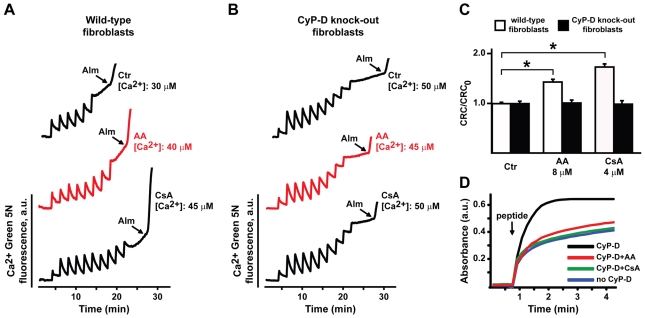
Effect of AA on PTP opening in wild-type or CyP-D knock-out mouse fibroblasts and on CyP-D isomerase activity. A, B, PTP opening of digitonized wild-type (A) or CyP-D knock-out (B) fibroblasts was measured with the whole-cell CRC assay. Experiments were started by the addition of digitonized cells (not shown) followed by pulses of Ca^2+^ (5 µM each) and are plotted as in Figure 1B. The use of AA is shown with a red trace. Alamethicin (Alm, 1 µM) was added at the end of each measurement to fully release Ca^2+^ from intracellular stores. C, the CRC/CRC_0_ ratio calculated as in Figure 1C indicates that both AA and CsA display an inhibitory effect on the PTP in wild-type, but not in CyP-D knock-out fibroblasts. Results are mean±SD of at least 4 experiments. We analyzed whether each pharmacological treatment increased mitochondrial Ca^2+^ uptake when compared to control conditions (Ca^2+^ uptake in the absence of the drug), and found a significant difference (Student's *t* test analysis; *: p<0.01) between the CRC of mitochondria treated with either AA or CsA, and the CRC of untreated mitochondria, indicating that each of these treatments inhibits the PTP in wild-type fibroblasts. D, isomerase activity assay (see Materials and Methods): after addition of the substrate peptide N-succinyl-Ala-Ala-*cis-trans*-Pro-Phe-p-nitroanilide (arrow), chimotrypsin cleaves the trans-isomeric form, causing a rise in absorbance. AA (8 µM) and CsA (8 µM) act as inhibitors of the enzymatic activity of CyP-D.

**Figure 5:**
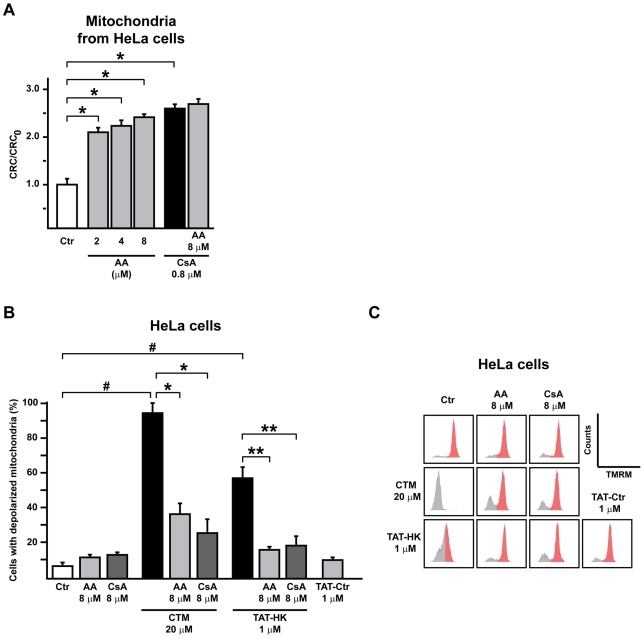
Effect of AA on mitochondrial potential of HeLa cells. A, bar graphs report the ratio between the CRC detected in the presence (CRC) and absence (CRC_0_) of increasing concentrations of AA in mitochondria from HeLa cells. PTP inhibition by AA and CsA is not additive. Results are mean±SD of at least 4 experiments. We analyzed whether, in mitochondria from HeLa cells, each pharmacological treatment increased Ca^2+^ uptake when compared to control conditions (Ca^2+^ uptake in the absence of the drug), and found a significant difference (Student's *t* test analysis; *: p<0.01) between the CRC of mitochondria treated with either AA (at various concentrations), or CsA, and the CRC of untreated mitochondria, indicating that each of these treatments inhibits the PTP. B,C cytofluorimetric analysis of mitochondrial depolarization. HeLa cells were incubated with the TMRM probe and treated for 1 hour with the reported concentrations of either clotrimazole (CTM) or TAT-HK peptide. An unrelated TAT-linked peptide (TAT-Ctr) is used as a negative control. Before the addition of either CTM or TAT-peptides, cells were preincubated for 30 minutes with CsA or AA, or with the CsA analogue cyclosporin H (in the dubbed Ctr conditions), to exclude for changes in TMRM signal unrelated to mitochondrial potential (see Methods). Bar graphs in B display the percentage of cells with depolarized mitochondria (mean±SD, n = 4). We analyzed whether each pharmacological treatment increased the percentage of HeLa cells with depolarized mitochondria when compared to control conditions (*i.e.*, percentage of cells with depolarized mitochondria in the absence of the drug), and found a significant difference (Student's *t* test analysis; #: p<0.01) between cells treated with either CTM or TAT-HK, and untreated cells, indicating that each of these compounds damages mitochondria. A similar pair wise comparison allowed to establish that pretreatment with either AA or CsA significantly decreased the percentage of cells with mitochondria depolarized by CTM (Student's *t* test analysis; *: p<0.01) or by TAT-HK (Student's *t* test analysis; **: p<0.01). Histograms in C report a representative experiment, where cells with polarized and depolarized mitochondria are indicated in red and grey, respectively.

The above data strongly suggested that AA could target mitochondrial CyP-D. To formally establish whether the interaction between AA and CyP-D determines PTP inhibition, we purified mitochondria from either wild-type or *Ppif^−/−^* (CyP-D null) mouse fibroblasts Figure 3A and [27]. First, we measured oxygen consumption, and we found that this is unaffected by AA both in wild-type and in CyP-D knock-out cells, as well as in mouse liver mitochondria (Figure 3A and data not shown). We then assessed CRC modulation. Consistent with results obtained on MLM, we found that AA inhibited the pore in mitochondria from wild-type fibroblasts, and that this effect was not enhanced by CsA (Figure 3B). Remarkably, AA was totally ineffective on mitochondria from CyP-D knock-out fibroblasts (Figure 3B). We confirmed these observations by CRC whole-cell experiments, *i.e.* by measuring mitochondrial Ca^2+^ retention capacity on digitonized cells (Figure 4A–C). We then repeated CRC experiments on human cervix adenocarcinoma HeLa cells, to address the issue of cell- and species-specificity. Our results confirmed those obtained in the mouse model, as AA inhibited the pore also in mitochondria isolated from HeLa cells, and the effect of AA did not add to that of CsA (Figure 5A). In addition, CyP-D was directly targeted by AA, as AA abrogated the peptidyl-prolyl *cis-trans* isomerase activity of purified CyP-D (Figure 4D).

### AA inhibits PTP-induced cell death

**Figure 6:**
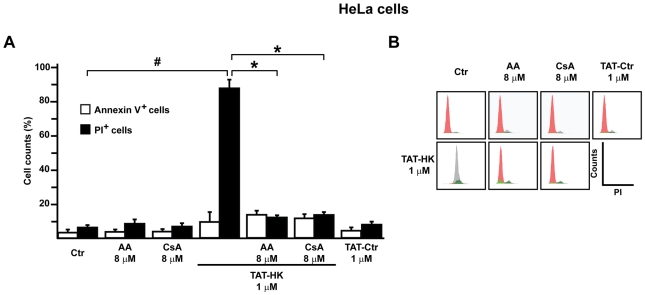
Effect of AA on HeLa cell viability. A, cytofluorimetric analysis of cell death induction in HeLa cells treated for 1 hour with TAT-HK. Where indicated, cells were preincubated for 30 minutes with CsA or AA. TAT-Ctr was used as a negative control. Cells were double stained with Annexin V-FITC and propidium iodide (PI), to respectively evaluate apoptosis induction as phosphatidylserine exposure on the cell surface (increased Annexin V-FITC staining) and cell death as plasma membrane permeabilization (increased PI staining). We considered Annexin V^+^ as early/intermediate apoptotic cells; PI^+^ as necrotic cells; double stained as late apoptotic cells. Bar graphs display the percentage of each cell population (mean±SD, n = 4; double positive cells are added to both bars). The number of PI+ cells was significantly increased by TAT-HK treatment (Student's *t* test analysis; #: p<0.01 when comparing untreated and TAT-HK treated HeLa cells). Moreover, HeLa cell pretreatment with either AA or CsA significantly protected from TAT-HK induced death (Student's *t* test analysis; *: p<0.01 when comparing TAT-HK treated HeLa cells pretreated or not with either AA or CsA). B, histograms reporting a representative experiment, where viable cells are indicated in red, PI^+^- and Annexin V^+^-cells are indicated in grey and green, respectively.

The potential pharmacological use of AA as a PTP modulator required to address whether the compound is active in whole cells. To this aim, we exploited the sensitivity of HeLa cells to PTP opening and to the ensuing apoptosis following hexokinase II (HKII) detachment from mitochondria with the drug clotrimazole (CTM) or with a HKII-displacing peptide (TAT-HK; [27]). We found that the marked mitochondrial depolarization induced by CTM or TAT-HK was similarly reverted by CsA or AA (Figure 5B,C). Moreover, HKII displacement from mitochondria with TAT-HK prompted a fast and dramatic cell death, mainly necrosis, as indicated by the low cell staining with the apoptosis marker Annexin V in the presence of a massive propidium iodide signal (Figure 6A,B). Either CsA or AA virtually abolished the necrotic response, whereas a small fraction of cells undergoing apoptosis was still detectable (Figure 6A,B), in line with the observation that modulating the intensity of the stress stimulus can switch the mode of cell death between apoptosis and necrosis [31]. A similar degree of cell death was observed when cells were treated with clotrimazole, and again either CsA or AA inhibited the process (data not shown).

## Discussion

The identification of PTP targeting drugs is a highly desirable result, as the PTP is involved in a wide range of diseases. By the use of CsA or of CyP-D knock-out animals it was established that dysregulated PTP induction is implicated in various forms of brain damage [32], [33] and neurodegenerative diseases [34], in muscular dystrophies caused by collagen VI deficiency [23], in hepatotoxicity, in ischemic injury of kidney, brain and heart and in cardiac ischemia/reperfusion damage [21], [22].

CyP-D is a promising pharmacological target. It is part of the CyP protein family, composed by ubiquitous and extremely conserved proteins located in different subcellular compartments [12], [35]. A first generation of CyP inhibitors (e.g. Debio-025 or NIM811) has overcome the problems connected to the immunosuppressant activity of CsA, caused by the formation of a CyP-A/CsA complex in the cytosol that inhibits lymphocyte activation, blocking the NF-AT transcription factor [36]. These molecules display promising clinical effects, as shown by their use in the treatment of some viral infections. Indeed, the chaperone activity of CyPs probably controls the correct folding of viral proteins. CyP-A interacts both with the HIV Gag protein and with the HCV NS5B polymerase, and both CsA and non-immunosuppressant CsA derivatives prevent HCV/HIV replication and are in phase I/II clinical trials [35]. In a different pathological setting, it is noteworthy that Debio-025 normalizes mitochondrial function and apoptotic rate on muscle samples derived from patients affected by Ullrich muscular dystrophy [37] and in a mouse model of the disease [38]. Nonetheless, these results are hampered by severe side effects of CsA derivatives, including nephrotoxicity, neurotoxicity, and hepatotoxicity [36], and by their poor permeability to the blood brain barrier [39]. Thus, a second generation of CyP inhibitors should demonstrate increased efficacy and improved safety profiles, and AA could be a lead compound in the search for new therapeutic molecules.

To the aim of selecting these more efficacious drugs, a fine comprehension of the interaction with their molecular targets is needed. The CsA-binding pocket of CyPs is formed by an extended groove where the isomerase active site is located. CsA half-inserts in the cavity, and a high binding affinity to CyPs requires the sum of multiple interactions: six CsA amino acids (residues 1–2 and 8–11) establish hydrogen bonds and hydrophobic contacts with 15 CyP amino acids. Residues 3–7 of CsA are exposed to the outside of the CyP-CsA complex and are prone to interact with other molecules [39]. In the case of AA, it was suggested that Phe6 and Phe9 are located in the region primarily affected by conformational variations [3], and a NMR study shows a high degree of flexibility when any of the two Phe residues is substituted with Gly [40]. We have found that substitutions in Phe6 or Phe9 abolish every effect on the PTP (see Figure 2B). Similarly, changes in these residues abrogate cell growth inhibition and cytotoxicity elicited by AA in tumour cell models [3], [40].

It must be highlighted that the biological activities of AA are possibly wider than the presently reported PTP inhibition. Indeed, both AA and CsA inhibit the PTP (present results), block cell uptake of the phallotoxin phalloidin ([8] and our unpublished data) and act as immunosuppressants [10], [11]. It is therefore highly probable that, similar to CsA, AA targets multiple cell CyPs. The CyP chaperone activity, *i.e.* the isomerization to the *cis* form of the peptidyl bonds preceding each proline [14], is involved in folding and association processes required for proteins to perform their functions [39]. Here we show that, similar to CsA, AA abrogates the peptidyl-prolyl *cis-trans* isomerase activity of CyP-D. This observation strongly suggests that the enzymatic inhibition of CyP-D is the molecular mechanism responsible for PTP inhibition. Thus, AA could have diverse biological effects, as CyPs modulate a variety of cell processes, including gene transcription, proliferation, survival, chemotaxis and motility, targeting molecules as diverse as the transcription factor NF-AT, MAP kinase upstream regulators [36], the membrane receptors CD147 and CXCR4 [39], [41], [42], and the kinases Itk [43], Crk [44] and Jak2 [45]. It must be highlighted that the impact of AA on cellular routines could be context-dependent, as different cell types utilize CyPs for a variety of processes under diverse conditions. An example of this complexity is provided by carcinogenesis. PTP inhibition contributes to the apoptosis resistance that characterizes neoplastic transformation [22], [46]. Therefore, a further PTP inhibition provided by AA should favor tumor growth. Accordingly, it was observed that CsA can enhance the progression of certain malignancies [47]. However, the issue of the CyP role in tumorigenesis is complicated by the observation that CyP-A is upregulated in a variety of tumor models, where it is involved in cancer cell survival, resistance to chemotherapeutics and metastasis [41], [48], [49], and that CsA treatment induces tumor necrosis and abrogates metastasis formation [45]. Moreover, CsA inhibits multidrug resistance proteins that are responsible for tumor chemoresistance [50].

It is known that AA abrogates the toxic effects of the phallotoxin phalloidin [8], [9]. We confirmed that AA inhibits membrane permeabilization by phalloidin (data not shown). However, we could not detect any effect of phalloidin either on mitochondrial Ca^2+^ retention capacity, or on mitochondrial potential (Figure S1). Therefore, phalloidin is inactive on the PTP, suggesting that AA counteracts its toxicity with a mechanism independent of pore inhibition, possibly antagonizing cell uptake of phallotoxins [8], [9].

In summary, we provide evidence that AA inhibits the mitochondrial PTP by targeting the peptidyl-prolyl *cis-trans* isomerase CyP-D, thus abrogating cell death caused by PTP inducers. AA could be exploited as a lead compound for the design of new CyP inhibitors, with implications for the pharmacological treatment of diverse pathological conditions.

## Materials and Methods

### Chemicals and cells

FITC-conjugated Annexin-V was from Boehringer Mannheim (Indianapolis, IN); Calcium Green-5N and tetramethylrhodamine methyl ester (TMRM) were from Molecular Probes (Eugene, OR); dinitrophenol was from Merck (Darmstadt, Germany); digitonin was from Calbiochem (San Diego, CA); all other chemicals were from Sigma (St. Louis, MO). Diaphragm adult fibroblasts were obtained by SV40 immortalization of primary cells from wild-type and *Ppif^−/−^* mice [27]; apoptosis inducers were added to exponentially growing cells in the absence of serum. Each experiment was repeated at least three times.

### Peptide synthesis

AA and four derivatives, Tyr9-AA, Gly9-AA, Gly6-AA, and octa-Gly9-AA were synthesized as reported elsewhere [3]. Briefly, the synthetic route was based on the preparation of three different fragments corresponding to the sequences 1–4 (containing *ab initi*o a C-terminal *tert*-butyloxycarbonyl-protected hydrazide moiety), 5–6 and 7–10. These fragments were subsequently assembled by the azide method following the Rudinger procedure obtaining the linear 5-4 sequence of AA. The same method was used for the cyclization reaction exploiting the possibility to separate the activation from the condensation which was carried out at concentration not exceeding 10^−3^ M in the presence of an inorganic base. Crude peptides were purified by elution on a Sephadex LH-20 (GE Healthcare Bio-Sciences, Uppsala, Sweden) column equilibrated and eluted with methanol-water (8∶2 v/v). Molecular masses of the peptides were confirmed by ESI-MS on a Mariner (PerSeptive Biosystem, Foster City, CA) mass spectrometer. The purity of the peptides, assessed by analytical reverse phase HPLC, was higher than 95%. The amino acid compositions of the peptide acid hydrolysates (6 M HCl, 22 h at 110°C in sealed evacuated vials) were determined with a 3A30 (Carlo Erba, Milan, Italy) amino acid analyzer. Peptides MIASHLLAYFFTELNbA-GYGRKKRRQRRRG (TAT-HK) and GYGRKKRRQRRRG-bA-EEEAKNAAAKLAVEILNKEKK (TAT-Ctr) were synthesized as described [27] by a solid phase method using an automatized peptide synthesizer (model 431-A, Applied Biosystems, Foster City, CA) and the fluoren-9-ylmethoxycarbonyl (Fmoc) strategy. HMPA PEGA resin (Novabiochem, Bad Soden, Germany) was used as solid support. Peptides were cleaved from the resins with a TFA/H20/thioanisole/ethanedithiol/phenol mixture. Crude peptides were purified by a preparative reverse phase HPLC. Molecular masses of the peptides were confirmed by mass spectroscopy with direct infusion on a Micromass ZMD-4000 Mass Spectrometer (Waters-Micromass). The purity of the peptides was about 95% as evaluated by analytical reverse phase HPLC.

### Isolation of mitochondria

Mitochondria were isolated either from livers of C57BL/6 mice through sequential centrifugations, or from cells, as described [27]. To obtain mitochondria, cells were disrupted with a glass-Teflon potter in a buffer composed by 250 mM sucrose, 10 mM Tris-HCl, 0.1 mM EGTA-Tris, pH 7.4. Nuclei and plasma membrane fractions were separated by a first mild centrifugation (700× *g*, 10 min), and mitochondria were then spinned down at a higher speed (7000× *g*, 10 min). All procedures were carried out at 0–4°C.

### Measurement of mitochondrial Ca^2+^ retention capacity

The Ca^2+^ retention capacity (CRC) assay was used to assess PTP opening following trains of Ca^2+^ pulses and measured fluorimetrically at 25°C in the presence of the Ca^2+^ indicator Calcium Green-5N (1 µM; λ_exc_: 505 nm; λ_em_: 535 nm; Molecular Probes). Experiments were performed either on isolated mitochondria or on whole cells [27]. Cells were washed in an isotonic buffer (130 mM KCl, 1 mM Pi-Tris, 10 mM Tris/Mops, and 0.1 mM EGTA/Tris, pH 7.4), and then permeabilized with 150 µM digitonin (15 min, 4°C), increasing EGTA to 1 mM. Digitonin was then eliminated and the number of cells carefully assessed before starting each experiment. Permeabilized cells or isolated mitochondria were placed in low (10 µM) EGTA in the presence of 2 µM rotenone/5 mM succinate, 10 µM cytochrome *c*, and Calcium Green-5N, which does not permeate mitochondria. Cells or mitochondria were then exposed to Ca^2+^ spikes, and fluorescence drops were used to assess mitochondrial Ca^2+^ uptake. PTP opening was detected as a fluorescence increase. Calcium Green-5N fluorescence was measured either with a fluorescence spectrometer LS50B (Perkin Elmer, Waltham, MA) or with a Fluoroskan Ascent FL fluorimeter (Thermo Electron Corporation, Waltham, MA).

### Western Immunoblot Analysis

Cell extracts were prepared at 4°C in 140 mM NaCl, 20 mM Tris·HCl (pH 7.4), 5 mM EDTA, 10% glycerol, and 1% Triton X-100 in the presence of phosphatase and protease inhibitors (Sigma). Samples were then denatured, separated in reducing conditions on SDS-polyacrylamide gels and transferred onto Hybond-C Extra membranes (Amersham, Little Chalfont, UK). Primary antibodies were incubated 16 hours at 4°C, and horseradish peroxidase-conjugated secondary antibodies were added for 1 hour. Proteins were visualized by enhanced chemiluminescence (Millipore, Billerica, MA).

### Mitochondrial respiration assay

Mitochondrial oxygen consumption was measured polarographically at 25°C with a Clark oxygen electrode (Yellow Springs Instruments, OH, USA). Mitochondria (0.6 mg per experimental point) were incubated in a solution of 130 mM KCl, 10 mM Tris-Mops, 1 mM Pi-Tris, 20 mM EGTA-Tris and 2 µM rotenone/5 mM succinate to assay basal respiration (state 4). ADP (200 µM) was subsequently added to measure state 3 respiration, followed by the uncoupling agent dinitrophenol (100 µM) to assess the maximal respiration rate.

### Flow cytometry analysis of mitochondrial depolarization and cell death induction

Flow cytometry recordings were performed as described [31], [51], [52]. Briefly, at the end of the incubation, cells were resuspended in 135 mM NaCl, 10 mM HEPES, 5 mM CaCl_2_ and incubated at 37°C in either TMRM (10 nM) to detect mitochondrial depolarization (reduced TMRM staining), or in FITC-conjugated Annexin-V and propidium iodide (PI, 1 µg/ml), to detect phosphatidylserine exposure on the cell surface (increased FITC-conjugated Annexin-V staining) and loss of plasma membrane integrity (PI permeability and staining). When TMRM was used to detect mitochondrial membrane potential, in each experiment one sample was treated with the protonophore FCCP (4 µM) as a positive control (full mitochondrial depolarization). Moreover, as TMRM can be pumped out of cells by multidrug resistance systems, and CsA is a multidrug resistance inhibitor, the CsA analogue cyclosporin H, which is inactive on the PTP, was used to block multidrug resistance in the absence of CsA [37]. Samples were analyzed on a FACSCanto II flow cytometer (Becton Dickinson, San Diego, CA, USA). Data acquisition and analysis were performed using FACSDiva software.

### PPIase Activity of CyP-D

The PPIase activity was assessed on recombinant CyP-D (10 ng per experimental point). The CyP-D cDNA was cloned in a pcDNA3 vector (Invitrogen, Carlsbad, CA) with a FLAG tag added at its 3′ end [22], and then purified by immunoprecipitation with a FLAG displacing peptide (Sigma). The enzymatic assay was performed following through a spectrophotometric analysis the rate of hydrolysis of N-succinyl-Ala-Ala-*cis-trans*-Pro-Phe-p-nitroanilide by chymotrypsin, as previously reported [53]. Chymotrypsin hydrolyzes only the *trans* form of the peptide, which is revealed as a rise in absorbance at 410 nM. The concentration of of the *cis* form is maximized by using a peptide stock dissolved in trifluoroethanol containing 470 mm LiCl, and its hydrolysis is limited by the rate of *cis-trans* isomerization.

## Supporting Information

Figure S1 **Effect of phalloidin (Ph) on mitochondria and cells.** A, ratio between the CRC detected in the presence (CRC) and absence (CRC_0_) of Ph (50 µg/ml) in MLM. The effects of CsA (0.8 µM) and AA (8 µM) are reported as positive controls. Results are mean±SD of 3 experiments. B, cytofluorimetric analysis of mitochondrial membrane potential after a 1 hour incubation with Ph (50 µg/ml). Treatment with the proton uncoupler FCCP (4 µM) is shown as a positive control of mitochondrial depolarization. Graphs report a representative experiments, where HeLa cells with polarized and depolarized mitochondria are indicated in red and grey, respectively. D is the percentage of cells with depolarized mitochondria.
(TIF)
