# Supplementary figures and images for: Antamanide, a Derivative of Amanita phalloides, Is a Novel Inhibitor of the Mitochondrial Permeability Transition Pore
Source: PLoS One. 2011 Jan 28;6(1):e16280. doi: 10.1371/journal.pone.0016280 (PMC3030572; doi:10.1371/journal.pone.0016280)

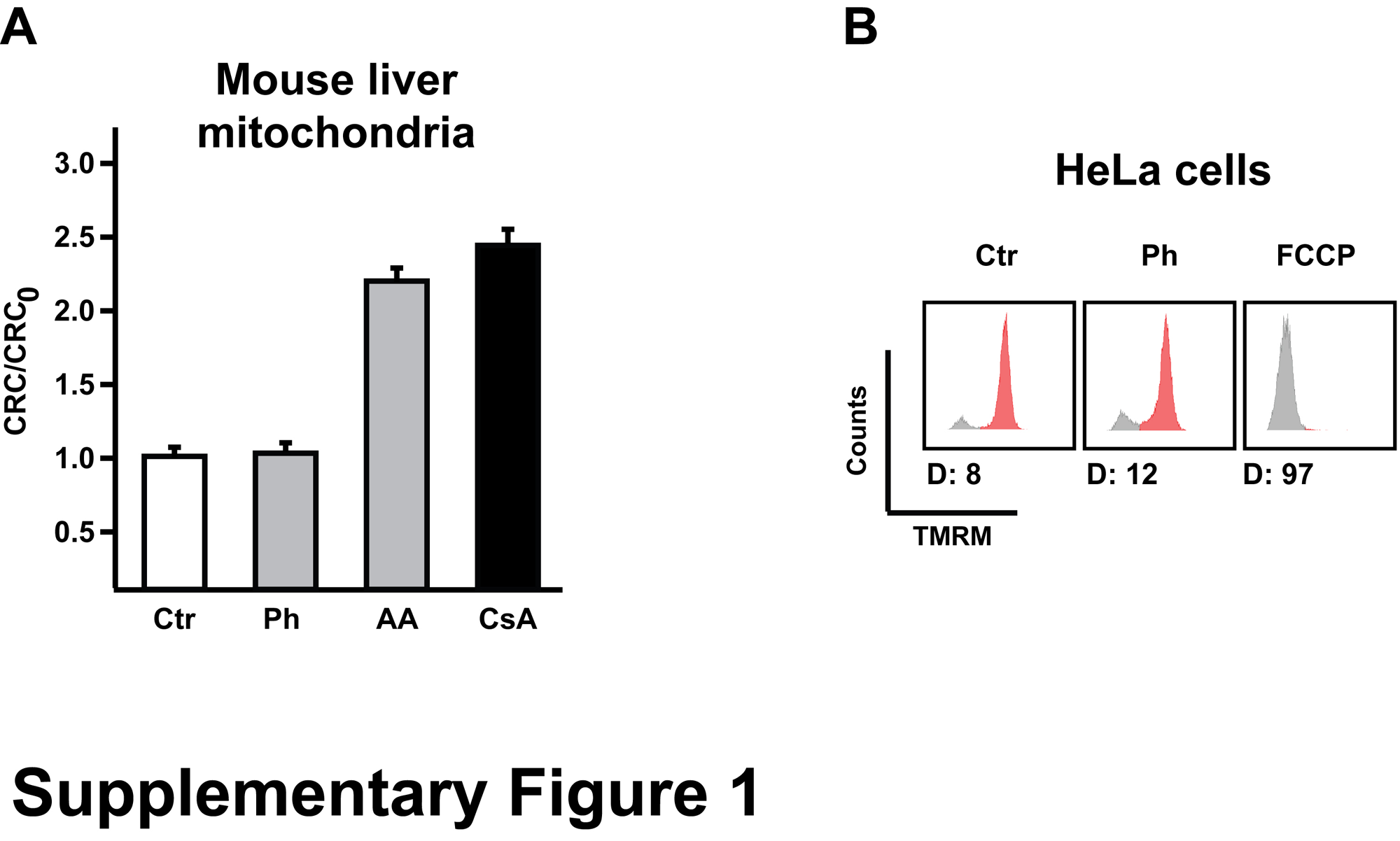

Supplement: Figure S1 — Effect of phalloidin (Ph) on mitochondria and cells. A, ratio between the CRC detected in the presence (CRC) and absence (CRC0) of Ph (50 µg/ml) in MLM. The effects of CsA (0.8 µM) and AA (8 µM) are reported as positive controls. Results are mean±SD of 3 experiments. B, cytofluorimetric analysis of mitochondrial membrane potential after a 1 hour incubation with Ph (50 µg/ml). Treatment with the proton uncoupler FCCP (4 µM) is shown as a positive control of mitochondrial depolarization. Graphs report a representative experiments, where HeLa cells with polarized and depolarized mitochondria are indicated in red and grey, respectively. D is the percentage of cells with depolarized mitochondria. (TIF) [file pone.0016280.s001.tif]
